# Economic Evaluation of Health Behavior Interventions to Prevent and Manage Type 2 Diabetes Mellitus in Asia: A Systematic Review of Randomized Controlled Trials

**DOI:** 10.3390/ijerph191710799

**Published:** 2022-08-30

**Authors:** Padam Kanta Dahal, Lal B. Rawal, Rashidul Alam Mahumud, Grish Paudel, Tomohiko Sugishita, Corneel Vandelanotte

**Affiliations:** 1 School of Health, Medical and Applied Sciences, Central Queensland University, Sydney, NSW 2000, Australia or; 2 Appleton Institute, Physical Activity Research Group, Central Queensland University, Rockhampton, QLD 4702, Australia; 3 Translational Health Research Institute (THRI), Western Sydney University, Sydney, NSW 2751, Australia; 4 NHRMC Clinical Trials Centre, Faculty of Medicine and Health, The University of Sydney, Camperdown, NSW 2006, Australia; 5 Section of Global Health, Division of Public Health, Department of Public Health, Tokyo Women’s Medical University, Tokyo 162-8666, Japan

**Keywords:** health behavior interventions, type 2 diabetes mellitus, Asian countries, economic evaluation, discount rate, cost effectiveness

## Abstract

Health behavior interventions implemented in Asian countries often lack economic evaluations that effectively address the problems of type 2 diabetes mellitus. This review systematically assessed the existing literature on economic evaluation of health behavior interventions to prevent and manage type 2 diabetes mellitus for people living in Asian countries. Eligible studies were identified through a search of six bibliographic databases, namely, PubMed, Scopus, Public Health Database by ProQuest, Cumulative Index to Nursing and Allied Health Literature Complete, Web of Science, and Google Scholar. Randomized controlled trials of health behavior interventions and studies published in the English language from January 2000 to May 2022 were included in the review. The search yielded 3867 records, of which 11 studies were included in the review. All included studies concluded that health behavior interventions were cost-effective. Eight of these studies undertook an evaluation from a health system perspective, two studies used both societal and health system perspectives, and one study utilized a societal and multi-payer perspective. This review identified the time horizon, direct and indirect medical costs, and discount rates as the most important considerations in determining cost effectiveness. These findings have implications in extending health behavior interventions to prevent and manage type 2 diabetes mellitus in low-resource settings, and are likely to yield the most promising outcomes for people with type 2 diabetes mellitus.

## 1. Introduction

Diabetes is a global public health problem that causes an estimated 1.6 million deaths each year [1,2]. The burden of diabetes has rapidly increased in the past 40 years, and it has been one of the major causes of years of life lost [3,4]. The International Diabetes Federation (IDF) reported that 537 million adults were living with diabetes in 2021, and this number is likely to reach 784 million by 2045 [5]. The IDF also indicated that over 75% of people with diabetes live in low- and middle-income countries (LMICs) [5], which constitute the majority of countries in Asia and Africa. The global economic burden of diabetes was USD 1.32 trillion in 2015 and is likely to reach USD 2.1 trillion by 2030, representing growth between the two years of 61% [3]. In 2021, diabetes resulted in health expenditure of at least USD 966 billion dollars, which represented a 316% increase over the past 15 years [5]. T2DM mellitus is the most common type of diabetes and accounts for 90–96% of all people with diabetes [5,6]. It is rising rapidly due to socioeconomic transition, rapid industrialization, and urbanization [7,8]. It is responsible for a heavy economic burden at individual, national, and global levels [9,10]. Similarly, the epidemiological trend of T2DM is projected to reach a pandemic level by 2030 in LMICs, and this may have a direct effect in the countries of Asia [11].

The majority (around 60%) of the people with diabetes live in Asia. This represents an estimated 323 million people, including 140.9 million people from China, 74.2 million from India, and 33 million people from Pakistan [5,12]. According to the IDF (2021), the total health expenditure associated with diabetes in the Asia region was approximately USD 278.52 billion [5]. In East Asia, South Asia, and the Pacific region, T2DM is already causing a heavy economic burden, which is likely to worsen if appropriate preventive measures are not taken [3]. Health care financing in most countries of Asia depends on out-of-pocket payments that create a high patient burden to access the proper health care services [13]. In recent years, most Asian countries have dedicated large amounts of funds to preventable diseases such as diabetes, yet they are still struggling to meet the cost of diabetes care [14,15].

Health behavior interventions prevent diseases and promote health [16]. They increase the individual level of self-care, happiness, and self-actualization, thus leading the way to optimal health and wellbeing [17]. Evidence shows that health behavior interventions are important to promoting healthy eating habits and regular physical activities, which are essential to reducing the risk of developing chronic health conditions such as diabetes, cardiovascular diseases (CVDs), hypertension, and obesity [16,18]. A study by Devaraj et al. documented that 94% of the participants made healthier food choices due to the influences of healthy diet intervention, and also improved their physical activity [19]. Thus, promoting health behavior is important to enhancing the quality of life and the health status of the population.

T2DM can be prevented by adopting healthy behaviors, which directly improve the quality of life and reduce the cost of health care [20]. A study by Dall et al. in 2015 reported that health behavior interventions have a large economic benefit and long-term effects on reducing the burden of T2DM [21]. Several reviews conducted in high-income countries reported that health behavior interventions, including those focusing on diet and physical activity, are cost-effective for the management and treatment of T2DM [22,23,24]. Health behavior interventions also reduce the probability of developing T2DM by 9.53% and have additional positive outcomes on life expectancy, quality-adjusted life year (QALY), and total cost savings over a lifetime [25,26,27,28].

However, although the majority of people with T2DM reside in Asian countries, these countries often lack health behavior interventions that effectively address the problem of T2DM. Moreover, of the limited number of studies conducted in this region, even fewer have conducted an economic evaluation of these interventions. This systematic review assessed the economic evaluation of health behavior interventions (i.e., diet, physical activity, tobacco use, stress management, drugs/alcohol consumption) that have been conducted to prevent and manage T2DM in Asian countries.

## 2. Materials and Methods

### 2.1. Design and Registration

This systematic review adhered to the Preferred Reporting Items for Systematic Reviews and Meta-analysis (PRISMA) guidelines [29]. PRISMA consists of a 27-item checklist and a 4-phase flow diagram used to improve the transparency in systematic reviews (the PRISMA checklist can be found in Supplementary Material). The protocol of this review has been registered with the International Prospective Register of Systematic Review (PROSPERO) database (Registration number: CRD42021249403), which helps to avoid duplication and mitigates opportunity reporting bias, as planned in the protocol.

### 2.2. Data Sources and Search Strategy

Literature searches were conducted in the six most relevant bibliographic databases, namely, PubMed, Scopus, Public Health Database by ProQuest, Cumulative Index to Nursing and Allied Health Literature (CINAHL) Complete, Web of Science, and Google Scholar. Appropriate medical subject headings (MeSHs), Boolean operators, Wildcards, and Truncation and Field tags were applied during the database search. The search included a combination of search terms related to: economic evaluation, health behavior intervention, T2DM, and Asian countries. The specific search terms for databases are available in Table 1. Searched articles were filtered by date, comparison of alterative intervention, English language, and peer review. Forward and backward reference searches of included articles were conducted to identify additional studies.

### 2.3. Eligibility Criteria

This study applied the following selection criteria:

Studies that included participants with T2DM and those with a high risk of developing T2DM due to obesity, impaired glucose tolerance, and/or impaired fasting glycaemia.

Randomized controlled trial (RCT) studies, published in the English language that examined any health behavior intervention components relevant to the prevention, treatment, and management of T2DM: diet, physical activity, tobacco use, stress management, drug/alcohol consumption.

Peer-reviewed studies conducted from 1 Jan 2000 to 31 May 2022 in Asia (i.e., all 49 countries listed by United Nations, including Russia) [30] and published in a peer-reviewed journal. The application of health economics in health promotion interventions was only established in 1998; hence, there were almost no studies published prior to 2000 [31].

Studies comparing one or more care alternatives against a health behavior intervention.

Studies that included an economic evaluation as defined by Drummond et al. [32], and assessed both the cost and effects of the health behavior intervention and the alternative intervention in terms of cost-effectiveness, cost–utility, cost–benefit, or cost-minimization analysis, and reported economic evaluation outcomes, such as QALYs, life year gained (LYG), direct cost, indirect cost, incremental cost-effectiveness ratio (ICER), cost–utility ratio, number needed to treat (NNT), or life expectancy.

Reviews, case reports, opinions papers, and articles published before 2000 were excluded from this review.

### 2.4. Study Screening and Selection

Padam Kanta Dahal (PKD) and Rashidul Alam Mahumud (RAM) conceptualized the study and developed the protocol. Lal B Rawal (LR), RAM, Grish Paudel (GP), Tomohiko Sugishita (TS), and Corneel Vandelanotte (CV) thoroughly reviewed the search strategy. The articles identified through the search were exported to the EndNote X9 software (Clarivate^TM^, Philadelphia, PA, USA), duplicates were removed, titles and abstracts were screened, and quality assessment was independently performed by PKD and GP. Full-text screening of the remaining articles was applied with the other authors (LR, RAM, TS, and CV). In the final stage, data extraction was performed by a single author (PKD).

### 2.5. Data Extraction and Quality Appraisal

A data extraction sheet for economic evaluation was developed based on Drummond [33] and the Consolidated Health Economic Evaluation and Reporting Standards (CHEERS) checklist [34] to collect all the relevant data for analysis. In the template, study characteristics (i.e., authors, study settings, country, intervention, target population, comparison, type of economic evaluation, estimated cost, outcome measures, and health behavior intervention components) and economic evaluation details (i.e., study perspective, cost-effectiveness measures, incremental cost-effectiveness ratio, cost–utility ratio, and net cost) were included for analysis. A comprehensive matrix was developed to summarize the study characteristics, type of economic evaluation, and the finding of the trial itself (i.e., behavior change achieved).

The Consensus Health Economic Criteria (CHEC) guidelines were employed to assess the quality of the studies included in this review [35]. The CHEERS checklist was used to review the standard of reporting the economic evaluation [34]. These checklists allowed assessment of each study’s strengths and weaknesses, a judgement to be made regarding the relevance of the findings, and optimization of the reporting of health economic evaluations [36,37].

### 2.6. Data Synthesis and Analysis

Data were tabulated and a narrative synthesis was conducted to illustrate the characteristics of the studies. Firstly, the studies included in this review were viewed through four elements of the synthesis process: (i) developing a theory about how the intervention works; (ii) developing the preliminary synthesis; (iii) exploring the relationship between the studies; and (iv) assessing the robustness of the synthesis [38]. Secondly, the studies were classified based on the health economic evaluation types, estimated costs, outcome measures, and health behavior intervention components. Lastly, costs were converted into US dollars and summarized using the Organization for Economic Co-operation and Development (OECD) exchange rates indicator [39].

The costs of the studies are reported based on the study’s perspective, time horizon, types of intervention, country situation, and targeted population. Costs are dependent on the perceived value, taxes, import duties, transportation cost, transaction cost, and market structure of the nation. Variations in cost estimations are based on the healthcare resource use, perspective, health system, continuous treatment process, and coordinated care model, covering both primary and post-acute hospital care. Diabetes treatment costs include medications, inpatient and outpatient care due to frequent laboratory tests, medications, and clinical supplies for patients who experience high out-of-pocket expenses. The costs of utilization of non-health care resources, such as transportation, productivity losses, informal care, household expenditure, and relocation and property losses, also contribute considerable expenses to the management of T2DM. When taking a health care perspective, estimated costs were explored in three categories, namely, direct medical costs, direct non-medical costs, and indirect costs. Direct medical costs refer to the costs of medical services that have a direct impact on health status, including consultation and specialist doctor fees, medicine costs, costs of diagnostic tests or imaging, hospitalization fees, costs of medical supplies (i.e., medical equipment, storage), and costs incurred when visiting healthcare providers and experts such as dieticians or endocrinologists. Direct non-medical costs refer to intervention costs and social services costs such as counseling costs, program evaluation costs, transportation costs, and associated food and accommodation costs when seeking health services [40,41,42,43]. When taking a societal perspective, indirect costs also refer to the community resource value costs, productivity losses, time spent by caregivers attending to the patient at the hospital/clinic, and potential loss of income [40,41,42,43,44]. Finally, a multi-payer perspective also takes into account the expenses to collect and pay for the services through multiple entities (such as health insurance companies or government rebates) [45].

In terms of cost-effectiveness scenario, ICERs were calculated, using the heuristic cost-effectiveness threshold proposed by the World Health Organization (WHO) and local thresholds (e.g., willingness to pay (WTP)). A WTP cost-effectiveness threshold is the highest amount policy makers (for example AUD 50,000 is considered to be the WTP for Australia) are willing to invest for a certain unit of health outcome. Interventions were considered to be cost-effective when the ICER was less than three times gross domestic product (GDP) per capita, as recommended by the WHO Choosing Interventions that are Cost-Effective (WHO-CHOICE) project [46], or when a WTP cost-effectiveness threshold exists for a specific country. Furthermore, the WHO developed three major decision rules, i.e., an intervention was recommended as (1) very cost-effective when the ICER per disability adjusted life year (DALY) averted is less than one times the GDP per capita, (2) cost-effective when the ICER per DALY averted one or more times the GDP per capita, but less than or equal to three 3 times the GDP per capita, and (3) not cost-effective when the ICER per DALY averted is more than 3 times the GDP per capita [46].

## 3. Results

**Figure 1:**
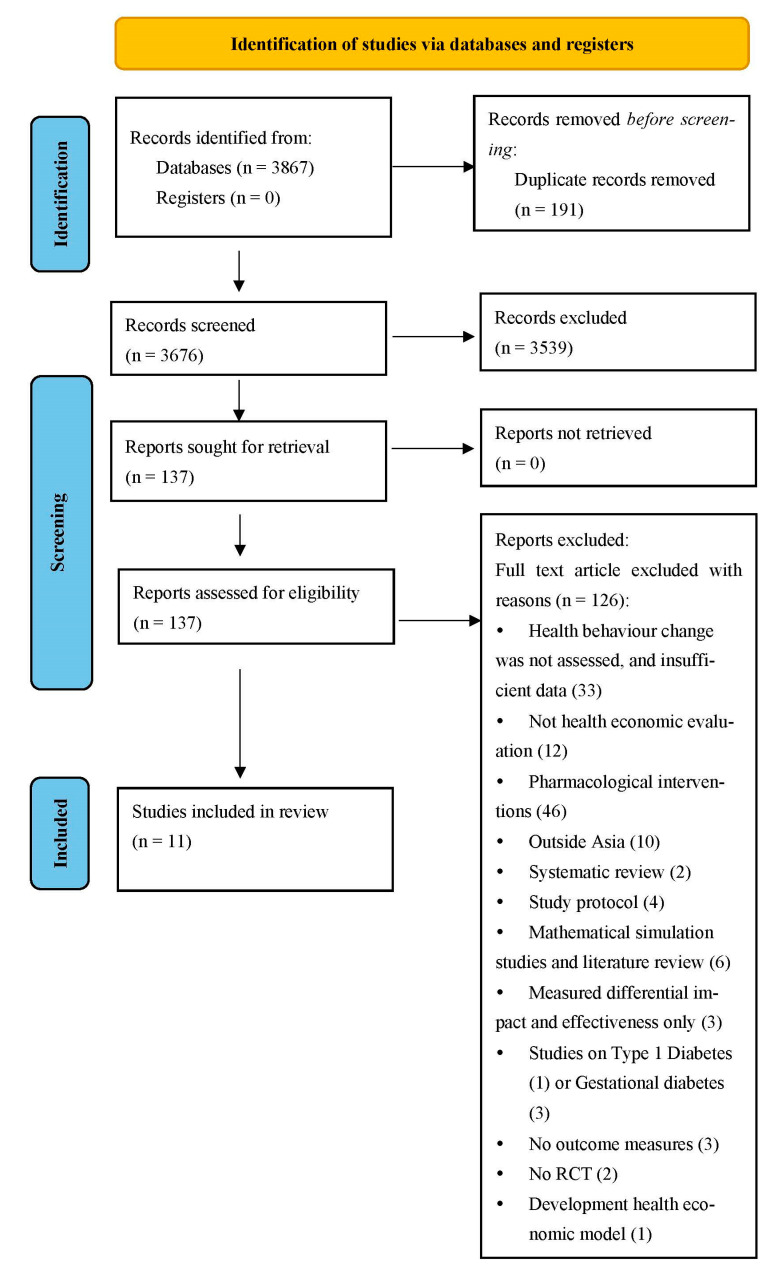
PRISMA flow diagram.

Initially, 3867 studies were identified from the six databases. A total of 3676 records remained after removing the duplicates. One hundred and thirty-seven full-text articles were assessed for eligibility after title and abstract screenings were conducted. However, no studies were identified through forward and backward searches. Finally, 11 articles met the selection criteria and were included in the review (see Figure 1 for the PRISMA flow diagram) [41,42,43,47,48,49,50,51,52,53,54].

### 3.1. General Characteristics of Selected Studies

The included studies were conducted in Bangladesh [48], China [47,52] including Hong Kong [51], India [41,43,49], Malaysia [54], Singapore [42,50], and Sri Lanka [53] with T2DM patients and participants who were at high risk of developing T2DM. Participants included in the studies were aged 5 years and above, and the average sample size of the studies was 920 participants (study samples ranged between 166 and 3539 participants). The studies used a Markov model [47,51,53,54], decision tree [42], or randomized controlled trial [41,43,48,49,50,51,52,53] to make a cost-effectiveness projection. The period for the potential effectiveness of the interventions was simulated from six months to a lifetime, and all studies performed a cost-effectiveness analysis of the health behavior interventions. Reported economic variables were costs, QALYs gained, DALYs averted, ICER, life expectancy, cases averted, willingness to pay, and NNT (Table 2). 

### 3.2. Descriptions of Interventions

Studies included in this review used various types of behavior change interventions. Two studies provided expert support to increase physical activity, promote healthy eating habits, reduce body weight, encourage tobacco cessation, reduce alcohol consumption, and ensure adequate sleep (Table 3) [41,50]. Two studies only focused on weight loss by increasing physical activity (i.e., walk for 30 min a day) [49], and improving dietary intake (i.e., reduce alcohol and carbohydrate intake, avoid sugar and inclusion of fiber-rich foods) [42,49]. Two studies used text messaging to change the behavior of participants [48,51], where participants either received text messages once a day for 6 months or received 66 messages over two years. One study implemented a mobile phone-based intervention for 1 year that provided behavior change suggestions, telephone follow-up, health education, diet and exercise monitoring, and peer support [52]. Another study provided 16 sessions of health behavioral counseling over 4 months and 8 sessions of maintenance classes over 2 months [43]. In one study, peer-educators delivered four face-to-face sessions annually on lifestyle modification for 3 years [53]. One study used six years of regular health screening (i.e., initial 2 h post-glucose test for 3 months, confirmatory diagnosis test, and annual physical examination) implemented by a health worker in a health center, and counseling on health behavior change at the 3-month follow-up with a physical examination up to one year later [47]. Finally, one study provided counseling on medication adherence, lifestyle modification, and self-glucose monitoring by a community pharmacist and family medicine physician every 3 months [54].

### 3.3. Thresholds

Nine studies used a cost-effectiveness threshold for decision making (i.e., deciding to promoting a health behavior over an alternative intervention) that reflected the maximum willingness to pay per unit of the health outcome [41,42,43,47,48,50,51,53,54]. Five of nine studies used a cost-effectiveness threshold decision rule proposed by WHO [41,42,43,48,53]. Three studies considered willingness to pay of values ranging between USD 4700.24 and 46,153 [47,50,54], and one study used the cumulative cost of delivering SMSs for 50 years (USD 3093) (Table 4) [51].

### 3.4. Cost of the Interventions

Eight studies considered the health system perspective that covered direct medical and direct non-medical costs [47,48,49,50,51,52,53,54]. These studies considered screening costs, health care service utilization costs, cost for delivering intervention, and resource costs. The majority of these studies obtained price data from patient’s self-reporting, hospital registers, prescriptions, and medical records. Three studies explored the costs from a combined health system and societal perspective, and one of these used a multi-payer perspective instead of a health system perspective [41,42,43]. In the studies that combined health care system and societal perspectives, direct and indirect medical costs and indirect costs of the interventions were estimated [41,42]. In the study that combined multi-payer and societal perspectives, costs for delivering intervention, costs for healthcare utilization, and direct non-medical costs were included; results showed that the societal perspective is slightly less cost-effective than the multi-payer perspective [43]. Health behavior intervention costs (according to both health care system and societal perspectives) in lower-middle-income countries, including India, Sri Lanka, and Bangladesh, were lower than those of high-income and upper-middle-income countries, including Singapore, Hong Kong, and China. Compared to the health care system perspective, the combination of societal and health care perspectives covered a larger number of participants in the community and a broader range of costs was covered (Table 4).

### 3.5. Cost Effectiveness of the Interventions

Health behavior change interventions to prevent and manage T2DM were cost-effective in comparison to the participants’ alternative care [41,42,43,47,48,49,50,51,52,53,54]. The decision to categorize an intervention as cost-effective was made based on the ICER and considered thresholds such as willingness to pay of between 1 and 3 times the GDP per capita in the study settings (minimum USD 165.21 and maximum USD 53,000.00). Based on the WHO recommended cost-effectiveness threshold, two studies [42,48] were considered as highly cost-effective and three as cost-effective [41,43,48,53]. The ‘Da Qing Diabetes prevention program’ in China was assessed as being cost-effective as it increased QALYs and cost saving of diabetes care over 30 years [47]. Five studies found that the health behavior modification interventions were cost-effective over a shorter time span (i.e., the average time span of these study was 1.8 years) [41,48,50,52,54]. The ‘stepwise approach for diabetes prevention program’ in India was likely to be cost-effective (i.e., mean QALYs gained 0.99 at a 95% CI: 0.018–0.179) with a 3-year time horizon and was expected to be cost-effective (threshold value USD 5748.37 (Int.$22,000) per QALY) with a long-term approach [43]. Health behavior modification and metformin interventions conducted in Singapore were considered to be highly cost-effective and worthy of implementing in a short time frame [42]. In this study, health behavior modifications, such as promotion of physical activities and dietary interventions, were implemented for 3 years and resulted in the gain of 2.03 QALY. Peer support interventions [41,53] and health behavior changes through physical activity and dietary modification [49] in developing countries such as India and Sri Lanka were deemed as being a cost-effective strategy for the management of T2DM. Education sessions focusing on T2DM self-management strategies through trained peer leaders helped to gain 1.65 overall QALYs [41] and avert 0.0017329 DALYs [53]. Intervention comprising health behavior modification, self-glucose monitoring, and medication adherence in Malaysia was deemed to be a cost-effective intervention from the health care system perspective, with 0.07 QALYs gained [54]. Most of the health behavior interventions conducted in high-income and upper-middle-income countries are likely to be highly cost-effective compared to those conducted in low-income and lower-middle-income countries (Table 4).

### 3.6. Quality Appraisal

All studies have a study perspective, a generalization (a clear viewpoint of analysis, and an indication of costs and outcomes that vary by location, setting, patient population, service provider, etc.), a time horizon, research questions, objectives, interventions, comparisons, and relevant costs that were measured and valued (measured in unit of local currency). Ten studies (approx. 90.91%) disclosed a conflict of interest and funding sources. Nine studies (81.82%) conducted probabilistic sensitivity analysis. However, eight studies (72.71%) did not adequately discuss ethical issues and four studies (36.36%) did not properly highlight the discount rate (Table 5).

## 4. Discussion

This systematic review assessed the economic evaluation of health behavior interventions being implemented among people with T2DM or at risk of developing T2DM living in Asian countries. This review included 11 studies that reported economic evaluation of health behavior interventions being conducted in community settings in six different Asian countries, namely, China, India, Bangladesh, Malaysia, Singapore, and Sri Lanka.

This review covered a wide range of studies that assessed the effectiveness of health behavior interventions to prevent and manage T2DM. From 11 studies, 2 provided expert support to increase physical activity, promote healthy eating habits, reduce body weight, encourage tobacco cessation, reduce alcohol consumption, and ensure adequate sleep; 2 studies focused on weight loss by increasing physical activity and improving dietary intake; 2 studies used text messaging to change the behavior of participants; 1 study implemented a mobile phone-based intervention; 1 study provided health behavioral counseling; 1 study examined peer-educators who delivered four face-to-face sessions; 1 study used regular health screening; and 1 study provided counseling on medication adherence, lifestyle modification, and self-glucose monitoring by a community pharmacist and family medicine physician for every 3 months. Among these interventions, weight loss programs (focusing on physical activity and diet), counseling for health behavior modification, and dietary text messaging were highly cost-effective compared to other health behavior interventions (i.e., promoting healthy diet, physical activity, tobacco cessation, and reduction in alcohol consumption, and one-on-one peer health education sessions). However, these other health behavior interventions were still cost-effective in general. As such, health behavior interventions are feasible in Asian countries to prevent and manage T2DM. A systematic review by Li et al. found that the interventions that used primary intervention approaches, such as intensive behavior modification, opportunistic screening for undiagnosed T2DM, intensive glucose control, smoking cessation, and annual screening, were very cost-effective [55]. The difference in the effectiveness of the interventions and their outcomes may be due to the good use of resources, quality of study data, and relatively good and resourceful health care settings. This review found that health behavior change interventions to prevent and manage T2DM were cost-effective in the short-term and likely to be cost-effective when applied over long-term time horizons; however, more data are needed to demonstrate long-term cost effectiveness. These findings are consistent with the findings of a previous systematic review focusing on high-income countries by Roberts et al. (2017), which identified cost effectiveness of behavioral interventions for T2DM [56]. This review highlighted the lack of evidence in relation to intensity, duration, format, and cost effectiveness of health behavior interventions. This suggests the need for long-term economic evaluation of health behavior interventions to fully understand the impact on development and treatment of T2DM [56]. Similarly, a systematic review focusing on South Asia by Singh et al. (2018) demonstrated that most interventions (i.e., primordial, primary, secondary, and tertiary prevention) used to control CVD and diabetes mellitus were cost-effective [57]. However, our study reported higher cost effectiveness of health behavior interventions than those reported by Singh et al. This may be because the study by Singh et al. considered the cost effectiveness based on observational, interventional, and decision models. Moreover, the focus of that study was on preventive and/or curative aspects of CVDs and any type of diabetes mellitus; however, our study solely focused on assessing the cost effectiveness of health behavior intervention implemented among people with T2DM. Therefore, we can argue that the health behavior interventions solely focused on people with T2DM may be more cost-effective when compared with the interventions that used combined approaches (i.e., preventive and/or curative strategies for CVDs and diabetes). Although three studies considered a societal perspective [41,42,43], two studies [41,42] combined this with a health care system perspective and one study [43] combined this with a multi-payer perspective. Our review showed that the combination of health care system and societal perspectives covered a larger number of participants for longer terms when compared to the health care system perspective [41,42,47,48,49,50,51,52,53]. As such, this may have a larger impact on communities and populations, possibly resulting in more cost savings; however, the conclusion was unclear due to the small number of included studies. Our findings are consistent with a previous systematic review conducted among the underserved population in US, which suggested that the cost of health behavior interventions to manage diabetes should be assessed from a societal perspective that includes all direct and indirect costs and can provide comprehensive cost-effectiveness analysis [58].

The majority of studies showed effectiveness in terms of cost per QALY gained, which is widely recognized and generally used to measure and compare the efficiency of interventions [41,42,43,47,48,51,54]. Similar findings were observed in a review from South Asia, where interventions were cost-effective due to the larger QALY gained [57]. Additionally, the review found that the QALYs gained by participants in the intervention group were consistently higher than those in the control group. This shows that health behavior interventions are more cost-effective than the alternatives, such as usual care, standard care, or pharmacological interventions. These findings are similar to those of a systematic review of economic evaluation studies conducted in high-income countries (the majority are US-based studies) to prevent T2DM [59]. Further, a review focusing on the cost effectiveness of health behavior interventions that included studies with a minimum 12 months follow-up among patients with diabetes reported improvement in life expectancy by 0.02–0.42 years and increased QALYs by 0.01–0.18 due to health behavior modifications [60].

The ICER is a summary measurement representing the value of intervention compared to an alternative and is considered a primary outcome of economic evaluations. This review found that health behavior interventions to prevent and manage T2DM appear to be cost-effective when ICERs were compared with cost per QALYs gained. Health behavior interventions were associated with higher effects and lower costs among the selected studies in the review and were under the threshold value that determines the cost effectiveness of health behavior interventions. A previous systematic review (studies included between 2008 and 2017) among individuals at high risk of T2DM reported that diabetes prevention programs (using health behavior interventions) are either cost-effective or cost-saving [61]. This review indicated that cost effectiveness of the health behavior interventions among the whole population needs further investigation, which may have great potential for managing T2DM. However, interventions to prevent T2DM in the UK using group-based education and peer support (including telephone contacts from volunteers) were unlikely to be cost-effective [27]. As such, our study shows a higher level of cost effectiveness of health behavior interventions for management of T2DM in Asian countries than those in other high-income countries, possibly due to the differences in health care resources, health care costs, and cost-effectiveness thresholds that exist for the specific country [56,62].

The overall quality of the selected studies was considered high and the findings were adequate to support the conclusions. However, the quality assessment did reveal some inadequacies regarding outcome identification, measurement, and ethical issues. The majority of the studies missed one or more reporting items of the CHEC list; however, some of the included studies were conducted prior to when several guidelines for conducting high-quality economic evaluation were developed [33,34,35]. Some studies did not describe appropriate outcomes, their valuations, or probabilistic sensitivity analysis, and/or disclose conflicts of interest and funding sources. Furthermore, most of the studies did not discuss the ethical and distributional issues (distributional implications of the population characteristics).

This review has some strengths and limitations. One of the strengths of this review is its in-depth search, for which a strong search string was applied that focused on the populations, interventions, comparisons, and outcomes. The study also used multiple databases (the PICOS framework), a forward/backward reference search, and a country-specific search to capture as many as possible of the relevant articles. Similarly, two reviewers reviewed titles and abstracts, and all authors screened the full text of the included studies. Furthermore, this review predominantly included high-quality economic evaluations and data were extracted based on the Drummond [33] and CHEERS [34] guidelines, which helped to increase the quality of the paper. However, this study was limited to the variation in countries’ geographic and socio-economic situations, which are inconsistent, as the resource input for the prevention and management of T2DM differs by country. For example, results of studies in Hong Kong and Singapore may not be similar to those in the low-resource setting of India and Bangladesh. Thus, the current results may not be generalizable to all low-income countries, and more studies are recommended based on similar settings and economic values. Further, cost-effectiveness evaluation based on the GDP-based threshold of 1–3 times GDP per capita may be misleading for country-level decision making due to the lack of a country-specific threshold [63]. The WHO assumption (i.e., 1 to 3 times GDP per capita) does not have a clear rationale or concrete evidence indicating that WTP for certain health gained is related to income; rather, it is likely to be affected by many factors such as resource use, data availability, and culture. Thus, study findings may not be similar. Moreover, the assumed GDP per capita as a threshold may be too high for LMICs, and studies using this threshold may be biased in the short term [64,65]. The length of studies was only from 6 months to 3 years, and some studies used modeling techniques for the long-term estimation of cost effectiveness. Hence, there is a need for long-term follow-up studies of health behavior change interventions. This is because the impact of health behavior changes and QALYs gained may not be appropriately reflected during the short-term period. Furthermore, modeling provides a prediction based on the current state and may not provide an explanation of the underlying situations, and assumptions may differ from reality. The majority of the included studies were funded by industry and may be more likely to report ICER below the thresholds. This may explain why all identified studies reported interventions as being cost-effective. As such, it is possible that publication bias occurred. Country-specific databases were excluded from the search due to language barriers; as such, some relevant studies may have been missed. Finally, the small number of included studies with an economic evaluation of health behavior interventions was another study limitation that may decrease the generalizability of the findings.

## 5. Conclusions

This review identified several health behavior interventions conducted in Asian countries that assessed cost effectiveness of the interventions to manage T2DM. However, the studies included in this review assigned a low priority to estimating costs from a societal perspective and there was no adequate information on the indirect cost saving of behavioral interventions for management of T2DM. This warrants the need to design and implement health behavior interventions that cover health systems and societal perspectives.

Given the importance of costs and health benefits of health behavior interventions for diabetes prevention and management, further studies, preferably of well-designed community-based intervention approaches, are essential to assess the longer-term impact of the interventions on disease outcomes and quality of life. For future research, it is important to also design, evaluate, and implement technology-based health behavior interventions in low-income community settings. Or for those who live in rural or remote geographical locations, as these interventions are able to reach many people at low cost. However, almost no studies have examined their cost effectiveness. Moreover, future research should consider economic evaluations of health behavior interventions in high-risk younger populations (i.e., those who have multiple risk factors for T2DM), which are lacking in the existing literature. Future studies should also implement a more rigorous study design, including randomized controlled trials. Finally, further studies should incorporate the impact of factors that influence individual behavior change, such as a country’s economic condition, environmental changes, production, storage, and supply of healthy foods, and individual susceptibility to T2DM, which may affect the sustainability of individual health behavior changes. This information is essential to guide health policy makers, planners, and program managers in efforts to prevent and manage T2DM and other NCDs.

## Figures and Tables

**Table 1:** Sample search terms for PubMed.

Concept	Key Words
Population	((type 2 diabetes mellitus[MeSH Terms] OR “Diabetes Mellitus” OR “Type 2 Diabetes” OR “impaired glucose” OR “insulin resistance” OR “non-insulin-dependent” OR “adult-onset diabetes”))
	AND
Intervention	((Lifestyle Intervention[MeSH Terms] OR Primary OR Secondary OR “Lifestyle Intervention” OR “Non-Pharmacological Interventions” OR “Community-based Intervention*” OR Behavioural OR “Randomised Control Trials” OR Diet OR “Physical Activit*” OR Tobacco OR Smoking OR Alcohol OR “Public Health Program”))
	AND
Comparator	Usual care OR Standard Care
	AND
Outcome	((Economic Evaluation[MeSH Terms] OR “Cost-effectiveness analysis” OR “Cost-utility analysis” OR “Economic evaluation” OR “Cost-benefit analysis” OR “Life year gained” OR “quality-adjusted life years” OR “disability-adjusted life years”OR “Incremental cost-effectiveness ratio” OR “Cost-utility ratio” OR “Sensitivity analysis” OR “Net cost*” OR “Health care cost” OR “health expenditure” OR “Budget impact analysis” OR “Cost consequences analysis” OR “Cost minimization analysis”))
	AND
Study type	Randomised controlled trial OR Controlled Trial OR RCT
	AND
Setting	((Asia[MeSH Terms] OR “Asia*” OR “South Asian” OR “Asian countries” OR “Southeast-Asia” OR Afghanistan OR Armenia OR Azerbaijan OR Bahrain OR Bangladesh OR Bhutan OR Brunei OR Burma OR Cambodia OR China OR “East Timor” OR Georgia OR “Hong Kong” OR India OR Indonesia OR Iran OR Iraq OR Israel OR Japan OR Jordan OR Kazakhstan OR Kuwait OR Kyrgyzstan OR Laos OR Lebanon OR Malaysia OR Mongolia OR Nepal OR “North Korea” OR Oman OR Pakistan OR “Papua New Guinea” OR Philippines OR Qatar OR Russia OR Saudi OR Arabia OR Singapore OR “South Korea” OR Sri Lanka OR Syria OR Taiwan OR Tajikistan OR Thailand OR Turkey OR Turkmenistan OR “United Arab Emirates” OR Uzbekistan OR Vietnam OR Yemen))

**Table 2:** General characteristics of the selected study.

Study and Year	Country	Population	Participant’s Age (Years) and Inclusions	Sample Size (Male + Female)	Variable of Interest	Time Horizon	Analytical Approach	Type of Economic Evaluation	Assumptions
Hu et al. (2020) [47]	China	People with IGT	Aged 25–74; IGT risk with T2DM	438	Direct medical costs; LE; QALYs; ICER	30 years; Lifetime	Markov model	Cost-effectiveness	Did not include non-medical costs or concern micro-vascular outcomes
Islam et al. (2020) [48]	Bangladesh	Patients with T2DM	Patients visited in hospital within 5 years; oral medication; phone access; able to read the text message	236 (male = 118 and female = 118)	Incremental health effects; QALYs; Incremental cost; ICER	6 months	Within-trial analysis	Cost-effectiveness	No information on uses of health care over the follow-up; power of study is insufficient to measure quality of life
Islek et al. (2020) [43]	India	Adults with IGT or IFG or both	Adult with overweight, obesity and IGT; IFG	578 (male = 364 and female = 214)	Cost; health benefits; ICERs, QALYs	3 years	Within-trial analysis	Cost-effectiveness	Cost was based on self-reported out-of-pocket expenses; costs per QALY gained were lower than expected
Li et al. (2021) [52]	China	Patients with T2DM	Patients with T2DM aged 18 years and above	215 (male = 142 and female = 73)	Cost; health benefits; ICERs	1 year	Within-trial analysis	Cost-effectiveness	Intervention short period; some potential costs were not included
Png et al. (2014) [42]	Singapore	Pre-diabetes people with risk of T2DM	People with risk of T2DM	2161	Costs; QALYs; ICER	3 years	Decision tree	Cost-effectiveness	Singapore GDP per capita is high, proportion of GDP spend is low; costs assumption may not be true
Ramachandran et al. (2007) [49]	India	People with IGT positive	Aged 35–55; IGT positive	531	Costs; number needed to treat (NNT)	3 years	Within-trial analysis	Cost-effectiveness	Cost of intervention was not evenly distributed over 3 years of study; quality of life not measured
Rosli et al. (2021) [54]	Malaysia	Participants with T2DM	Aged 18 and above	166	Costs; QALYs, ICER	6 months	With-in trial analysis and Markov model	Cost-effectiveness	RCT relies on single setting and only payer perspective
Sathish et al. (2020) [41]	India	People with high risk of diabetes and IGT	Aged 30–60	1007	Costs; QALYs; ICER; WTP	2 years	Within-trial analysis	Cost-effectiveness	Provide knowledge in LMIC; short follow-up; suspected recall bias
Shearer et al. (2021) [53]	Sri Lanka	Young and adults with T2DM risk	Aged 5–40 years	3539	Cost; health benefits; ICERs, DALYs	3 years	Within-trial analysis and Markov model	Cost-effectiveness	Lack of unit cost for diabetes complication; unable to include overhead cost
Siaw et al. (2018) [50]	Singapore	High risk of uncontrolled diabetes	T2DM patients	330	Costs; QALYs; ICER	6 months	Within-trial analysis	Cost-effectiveness	Long-term impact cannot be explored; patients with uncontrolled diabetes may not be generalizable to patients with good glycemic control
Wong et al. (2016) [51]	Hong Kong	People with IGT	People with high risk of T2DM	Not available	Costs; QALYs; ICER	2 years and 50 years	Within-trial analysis and Markov model	Cost-Effectiveness	Some clinical data were adopted from DPP and DPPOs; need more study subjects; did not account for health state

T2DM: Type 2 diabetes mellitus; IGT: impaired glucose tolerance; IFG: impaired fasting glycemia; GDP: gross domestic product; DALYs: disability-adjusted life years, QALYs: quality-adjusted life years; ICER: incremental cost-effectiveness ratio, WTP: willingness to pay; RCT: randomized controlled trial; NTT: number needed to treat; LE: life expectancy.

**Table 3:** Details of health behavior interventions to manage T2DM and comparisons.

Study and Year	Health Behavior Interventions to Manage T2DM	Comparisons
Hu et al. (2020) [47]	Six years of therapeutic lifestyle, regular health screening services by health worker in a health center; lifestyle counseling at 3-month follow-up with physical examination up to one year later.	Pre-diabetes management without intervention
Islam et al. (2020) [48]	Text messages were sent on the principals of behaviour learn theory; participants received 90 text messages randomly once a day for 6 months.	Standard care for glycemic control for T2DM patients
Islek et al. (2020) [43]	Provided 4 months (16 sessions) of behavioral counseling; 2 months (8 sessions) of maintenance class with 3 years follow-up.	Standard care-single day 1-on-1 visit with health care professionals; 1 group class on diabetes preventions
Li et al. (2021) [52]	Provided suggestions, telephone follow-up, health education, diet, exercise, monitoring, and peer support using mobile phone for one year.	Standard medical care
Png et al. (2014) [42]	Weight loss through increase in physical activity and dietary modification for 3 years.	Metformin treatment; placebo
Ramachandran et al. (2007) [49]	Participants were asked to walk briskly at least 30 min a day; reduction in total calories, and refined carbohydrates and fats, avoidance of sugar, and inclusion of fiber-rich foods for 3 years.	250 mg metformin a day; usual clinical diabetes care
Rosli et al. (2021) [54]	Provided tailored counseling on medication adherence, lifestyle modification and self-glucose monitoring by community pharmacist and family medicine physician every 3 months (i.e., baseline, 3 months, and 6 months), which lasted 20–45 min.	Routine diabetes care and treatment
Sathish et al. (2020) [41]	Total of 15 group sessions delivered in 12 weeks conducted in the community on Saturday and Sunday; experts in nutrition, diabetes, and physical activities provided 2 half-day session on diabetes management; trained peer leader provided 12 sessions; objective was increase physical activities, promote healthy eating habits and tobacco cessation, reduce alcohol consumption, reduce body weight, and ensure adequate sleep. Intervention was conducted for 1 year.	Provided usual diabetes care with health education booklet
Shearer et al. (2021) [53]	Provided four one-on-one sessions annually with trained peer educators who provided individualized lifestyle modification advice.	Provided one annual one-on-one session with trained peer educators
Siaw et al. (2018) [50]	Physician referred to diabetes nurse educators or dieticians; clinical pharmacist follow-up every 4–6 weeks, via face-to-face meetings or phone calls of at least 20–30 min. The intervention duration was 6 months.	Usual care with referral to diabetes nurse educators or dieticians
Wong et al. (2016) [51]	SMS to prevent onset of T2DM in addition to usual clinical practice for 2 years.	Usual clinical practice

T2DM: T2DM mellitus; SMS: Short Message Service.

**Table 4:** Economic evaluation details of the health behavior interventions.

Study and Year	Study Perspective	Costs	Currency and Discount Rate (%)	Cost Reported Rate and Year	Unit of Cost-effectiveness Measure	Incremental Cost Effectiveness Ratio (ICER)	Conclusion or Recommendation	Threshold
Hu et al. (2020) [47]	Health care system	* Intervention costs for 30 years- CNY 74,510 (USD 11,698.07); Lifetime intervention costs—CNY 86,294 (USD 13,548.16)	Chinese Yuan; 3%	USD 1 = CNY 6.37 in 2021	QALYs	* 30 years- CNY −8211 (USD −1289.13) costs per QALY; Lifetime- CNY −1652 (USD−259.36) cost per QALY	Highly cost-effective	WTP (* USD 5787.64 (CNY 37,446) per QALY)
Islam et al. (2020) [48]	Health care system	Total cost-** Int. $2842; Cost per participants-** Int. $24	Taka; Not applied	International dollar in 2013	QALYs	** Int. $2406 costs per QALY	Highly cost-effective	GDP and WTP (** Int. $7120 per QALY for 2015)
Islek et al. (2020) [43]	Multi payer and societal	Direct medical costs to intervention-*** Int. $959; Cost to health care utilization-*** Int. $125; Direct non-medical costs to intervention-*** Int. $1438; Direct cost to screening-*** Int. $681; Direct non-medical cost to screening-*** Int. $28 (Total-*** Int. $3231)	INR; 5%	Int. $1 = INR 18.4 in 2019	QALYs	Multi payer-*** Int. $8107 cost per QALY gained; Societal-*** Int. $12,099 cost per QALY gained	Cost-effective	GDP and WTP (*** US$ 5748.37 (**** Int. $22,000) per QALY)
Li et al. (2021) [52]	Health care system	* Health costs—CNY 1169.76(USD 183.55) per year per patients; Usual care costs—CNY 1775.44 (USD 278.74) per patients per year	Chinese Yuan; Not mentioned	USD 1 = CNY 6.37 in 2021	Control rate of AbA1c	* ICER- CNY −22.02 (USD −3.45) per patients per year	Cost-effective	Not provided
Png et al. (2014) [42]	Societal; Health care system	Total cost in health system perspective USD 8896Total societal cost for diabetes patients USD 28,447	Singapore dollar; 3%	USD 1 = SGD 1.25 in 2012	QALYs	ICER of lifestyle intervention compared to Placebo-USD 17,184 per QALY	Highly cost-effective	GDP (USD 53,000)
Ramachandran et al. (2007) [49]	Health care system	Direct medical costs—USD 117 per participants; Total lifestyle modification costs—USD 225	INR; Not discounted	USD 1 = INR 45.11 in 2006	NNT	Incremental cost-effectiveness ratio-USD 1052	Cost-effective	Not provided
Rosli et al. (2021) [54]	Health care system	Mean intervention cost: USD 28.64 per participants	Ringgit Malaysia (RM); 0 for six months and 3% for lifetime	USD 1 = MYR 4.241 in 2019	QALYs	ICER—USD 280.79 per QALY gained	Cost-effective	USD 4700.24–6714.62
Sathish et al. (2020) [41]	Health care system; Societal	Total intervention cost—USD 12,096 (USD 24.2 per participant); Health system perspective average cost per participant—USD 306.6; Societal average cost per participant—USD 367.8	INR; 3%	USD 1 = INR 68.4 in 2018	NNT and QALYs	For health system perspective ICERs—Dominance to USD 276.1; Societal-ICERs—USD 114.2 to 476	Cost-effective	GDP and WTP (USD 2036)
Shearer et al. (2021) [53]	Health care system	Intervention group costs—USD 69.95; Control group costs—USD 69.03	Sri Lankan Rupee; 3%	USD 1 = LKR 148 in 2017	DALYs	ICER-USD 2316.48 per DALY averted	Cost-effective	GDP (1- and 3-times Sri Lankan GDP per capita) (USD 9185.64)
Siaw et al. (2018) [50]	Health care system	Direct medical costs for interventions: USD 535.47 and for control: USD 601.50	Singapore dollar; Not applied	USD 1 = SGD 1.25 in 2014	Change is HbA1c level	Dominated	Cost-effective	WTP (USD 165.21-USD 5000 per improvement in glycemia)
Wong et al. (2016) [51]	Health care system	Average costs for 2 years for intervention—USD 342.94 per patient and for control—USD 461.33; For 50 years, average cost for intervention—USD 12,107.40 and for control USD 12,958.17	Hong Kong dollar (HKD); 3%	USD 1 = HKD 7.8 in 2011	QALYs	Incremental per QALY—0.071	Cost-effective	50 years cumulative cost of SMS group (USD 3093.78)

INR: Indian Rupees; CI: confidence interval; CVD: cardiovascular disease; QALYs: quality-adjusted life years; ICER: incremental cost-effectiveness ratio, RCT: randomized controlled trial; NTT: numbers needed to treat; LY: life year; * Average exchange rate for Chinese Yuan (CNY) to USD on 30 Dec 2021 was 6.373; ** International dollar adjusted estimated purchasing power parity (PPP) conversion factor for Bangladesh in 2013; *** Average exchange rate of Indian Rupees to USD in 2021 was 70.420; **** International dollar applying Indian price inflation and PPP conversion for 2019 (Int. $1 = INR 18.4); USD: United States Dollar; SGD: Singapore Dollar; HKD: Hong Kong Dollar; LKR: Sri Lankan Rupee.

**Table 5:** Quality assessment using the CHEC check list.

No.	CHEC List	Number of Studies Satisfying	Percentage
1	Is the study population clearly described?	11	100
2	Are competing alternatives clearly described?	11	100
3	Is a well-defined research question posed in answerable form?	11	100
4	Is the economic study design appropriate to the stated objective?	11	100
5	Is the chosen time horizon appropriate in order to include relevant costs and consequences?	11	100
6	Is the actual perspective chosen appropriate?	11	100
7	Are all important and relevant costs for each alternative identified?	11	100
8	Are all costs measured appropriately in physical units?	11	100
9	Are costs valued appropriately?	11	100
10	Are all important and relevant outcomes for each alternative identified?	9	81.82
11	Are all outcomes measured appropriately?	11	100
12	Are outcomes valued appropriately?	11	100
13	Is an incremental analysis of costs and outcomes of alternatives performed?	11	100
14	Are all future costs and outcomes discounted appropriately?	10	90.91
15	Are all important variables, whose values are uncertain, appropriately subjected to sensitivity analysis?	9	81.82
16	Do the conclusions follow from the data reported?	11	100
17	Does the study discuss the generalizability of the results to other settings and patient/client groups?	11	100
18	Does the article indicate that there is no potential conflict of interest of study researcher(s) and funder(s)?	10	90.91
19	Are ethical and distributional issues discussed appropriately?	8	72.71

## Data Availability

Not applicable.

## Supplementary Materials

The following supporting information can be downloaded at: https://www.mdpi.com/article/10.3390/ijerph191710799/s1, Supplementary File S1: PRISMA 2020 Checklist.
